# Immune modulation of some autoimmune diseases: the critical role of macrophages and neutrophils in the innate and adaptive immunity

**DOI:** 10.1186/s12967-017-1141-8

**Published:** 2017-02-15

**Authors:** Kely Campos Navegantes, Rafaelli de Souza Gomes, Priscilla Aparecida Tártari Pereira, Paula Giselle Czaikoski, Carolina Heitmann Mares Azevedo, Marta Chagas Monteiro

**Affiliations:** 1 Pharmaceutical Science Post-Graduation Program, Health Science Institute, Federal University of Pará/UFPA, Belém, PA 66075900 Brazil; 2 Department of Clinical, Bromatological and Toxicological Analysis, Ribeirão Preto Pharmaceutical Sciences School, USP-SP, Ribeirão Preto, SP Brazil; 3 College of Pharmacy, Guairacá Faculty-PR, Guarapuava, PR Brazil

**Keywords:** Macrophages, Neutrophils, Autoimmunity, Autoimmune disease, Inflammation

## Abstract

Macrophages and neutrophils are key components involved in the regulation of numerous chronic inflammatory diseases, infectious disorders, and especially certain autoimmune disease. However, little is known regarding the contribution of these cells to the pathogenesis of autoimmune disorders. Recent studies have aimed to clarify certain important factors affecting the immunogenicity of these cells, including the type and dose of antigen, the microenvironment of the cell-antigen encounter, and the number, subset, and phenotype of these cells, which can prevent or induce autoimmune responses. This review highlights the role of macrophage subsets and neutrophils in injured tissues, supporting their cooperation during the pathogenesis of certain autoimmune diseases.

## Background

Epithelial and mucosal barriers, natural antimicrobial products, immune cells, pattern-recognition receptors, and soluble products, cytokine and opsonins (e.g., complement) are critical innate components. In this context, neutrophils and macrophages play an important role in induction either pro-inflammatory or anti-inflammatory responses into the inflammatory site [1, 2]. Thus, these cells are key components involved in the development of inflammatory responses of diverse pathological conditions, such as chronic inflammatory diseases, infectious disorders, autoimmunity and others diseases [3–5]. Autoimmunity reflects an imbalance between effectors and regulatory mechanisms, including the defective elimination and/or control of innate and adaptive responses and the activation of cells with of varying subsets and phenotypes, such as macrophages and neutrophils, which release several products into tissue. Thus, this review highlights the role of macrophages subsets and neutrophils in the peripheral tissues, and also further supporting their cooperation during the development of the pathogenesis of T cell-mediated autoimmune disease, as type 1 diabetes mellitus and rheumatoid arthritis.

## Macrophages and neutrophils: development and inflammation

### Origin and development of neutrophils and macrophages

**Fig. 1:**
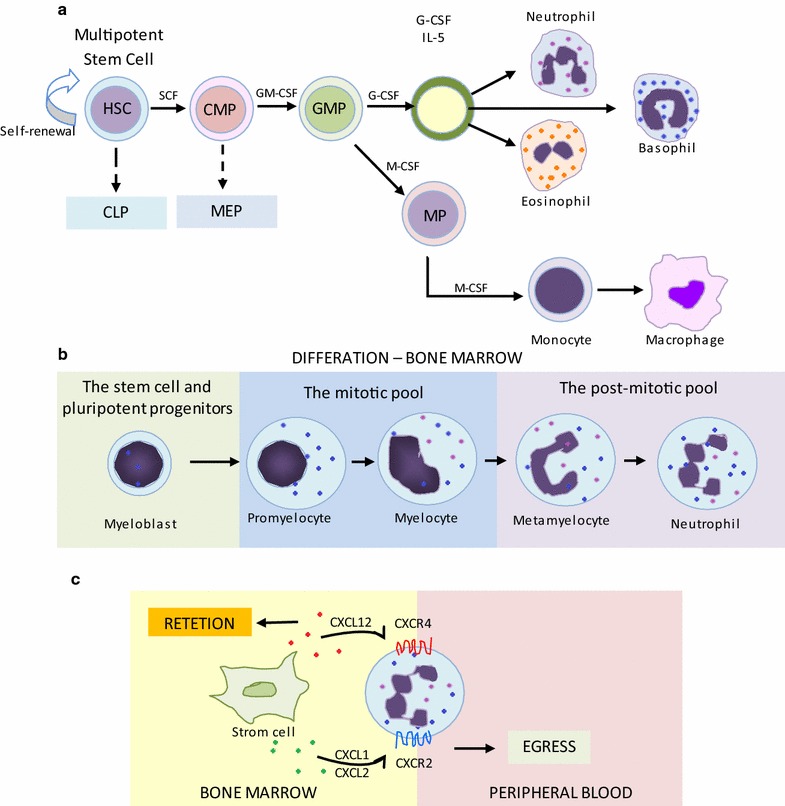
Origin and development of macrophages and neutrophils. **a** The generation of macrophages is dependent on hematopoietic growth factor receptor Csf1r (c-fms, M-CSFR, CD115). The known ligands of Csf1r, Csf1/M-CSF and interleukin (IL)-34 are likely both important for the development of the mononuclear phagocyte lineage. On the other hand, hematopoietic cytokines as granulocyte–macrophage colony-stimulating factor (GM-CSF) and granulocyte colony stimulating factor (GCSF) that promote neutrophil progenitor proliferation and differentiation. **b** Bone marrow neutrophil lineage cells can be divided into three compartments: the stem cell pool (stem cells and pluripotent progenitors), the mitotic pool and the post-mitotic pool. **c** The regulation of Neutrophil egress from de bone marrow by CXCR4 and CXCR2 chemokine ligands, where stromal cells produce C-X-C-motif chemokine ligand (CXCL) 12 that binds to C-X-C-motif chemokine receptor (CXCR) 4, leading to neutrophil retention, while release is mainly mediated by CXCR2. Hematopoietic stem cell (HSC), common myeloid progenitor (CMP), granulocyte–macrophage progenitor (GMP), myeloid progenitor (MP)

The first lines of defence against pathogens are the phagocytes cells, in which macrophages and neutrophils are included [6]. Neutrophils, the very short-lived human white blood cells (8–12 h in the circulation and 1–2 days in tissues), are the most abundant leukocytes in blood playing a primary role in the innate immunity [7]. These cells are produced in the bone marrow from multipotential progenitor cells, under the action of numerous mediators in particular growth factors called granulocyte colony-stimulating factor (GCSF), which are the main regulator of the granulocytopoiesis as shown in Fig. 1 [8–10]. The most immature cell of the granulocytic lineage is known as myeloblast. The proliferation and differentiation of these progenitors and these cells depend on hematopoietic cytokines such as GCSF, gene expression (responsible for the formation of granular proteins involved in cell function), myeloid transcription factors, forming the myeloid phagocyte system (MYPS) [8, 11, 12].

Thus, the GCSF acts by binding GCSF receptor, a family member of the class I cytokine receptor, promoting the neutrophil’s population life cycle that includes its proliferation, differentiation, releasing of mature cells from the bone marrow and survival [9, 10, 13, 14]. In this context, the bone marrow neutrophil’s population can be distributed in the steam cell pool, the mitotic pool and the post-mitotic pool [10, 14]. The first to mature are the hematopoietic stem cells and pluripotent progenitors; the next population, the mitotic pool, is composed of granulocytic progenitor cells such as myeloblasts, promyelocytes, and myelocytes. Lastly, mature neutrophils (metamyelocytes) are part of the post-mitotic pool, which constitutes the major source of neutrophils that can be easily mobilized and rapidly recruited to sites of infection [10, 14, 15].

All these populations are in homeostasis that includes a well-preserved equilibrium among granulopoiesis, bone marrow storage and release, intravascular transit, and destruction [14]. Therefore, after the neutrophil production, development and storage in the bone marrow, its releasing includes a transcellular migration from the sinusoidal endothelium to the circulation [14, 16]. Then, among existing mechanisms, the chemokines and their receptors play a key role about the balance between neutrophil release and retention. The major role is played by the stromal derived factor-1 (SDF-1) produced in the bone marrow and its ligation with the C-X-C motif chemokine receptor (CXCR) types 2 and 4. While the interaction of SDF-1 with the CXCR2 leads to the release of neutrophils, the interaction with the CXCR4 produces the opposite effect, leading to the retention of the neutrophils in the post-mitotic pool (Fig. 1b) [10, 17, 18].

After the bloodstream, the neutrophil migrate into the tissues to perform its function. However, near the inflammatory lesions neutrophils adhere to the endothelial wall, leave the blood vessels and actively migrate into the inflammatory focus [10]. This type of cells without external stimuli dies by apoptosis [12]. The process of cells death is a natural endpoint that occurs when the plasma membrane lose its integrity, or in the presence of cell fragments into discrete bodies and in the engulfment of the cell by another [19, 20]. Thus, to maintain the immune homeostasis is necessary the clearance of the apoptotic neutrophils by macrophages [14, 15]. This process is called “efferocytosis” and involves the liver X receptor (LXR), the decreasing in the production of interleukin (IL)-23, IL-17 and GCSF, whereas its weakened clearance has been associated with autoimmune diseases [10, 21, 22].

The other major defence against pathogens, inflammatory diseases and autoimmune diseases are the macrophages [23]. This type of leukocyte has its origin in hematopoietic stem cell with a myeloid progenitor forming the mononuclear phagocyte system (MPS) as shown in Fig. 1 [24]. Cytokines, as the macrophage-colony stimulating factor (M-CSF) and hematopoietic growth factor receptor (Csf1) expressed in monocytes, macrophages, mononuclear phagocyte precursors, are the main regulators of the MPS and both important for the development of phagocytic lineage [24, 25]. Therefore, MPS is constituted by monocytes, macrophages and dendritic cells (DC) [24]. Monocytes, precursor of tissue macrophages, are present in the bone marrow, circulation, and spleen, while the macrophages resides in lymphoid and non-lymphoid tissues [24]. However, recent studies suggest that many adult tissue macrophages have their origins during the embryogenic development, not only during the adult phase [26–28].

Thus, the embryonically derived macrophages are firstly detected and developed in the yolk sac being the only leukocyte produced independently of monocytes [29]. Afterward, all immune lineages forming the definitive hematopoietic stem cells (HSCs) that migrate to the foetal liver [27]. The principal site of haematopoiesis just became the bone marrow in the perinatal period [30]. These embryonic—resident tissue macrophages along the adulthood loss the capacity to identify macrophage populations [27]. Then, even with an origin dependent or not of monocytes, macrophages contribute to the homeostasis of the immune system.

### Activation of macrophages and neutrophils

**Fig. 2:**
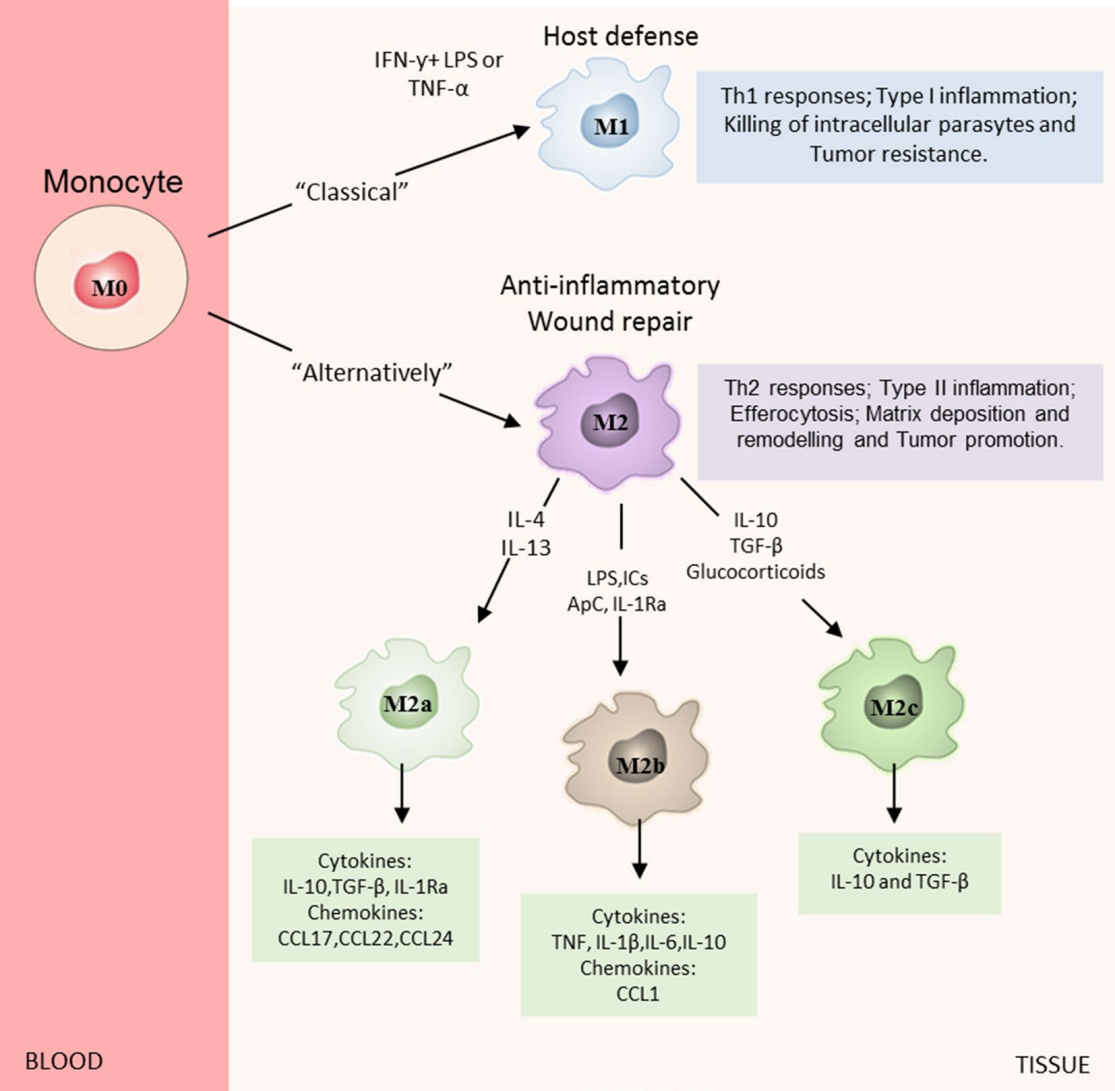
Schematic representation of macrophage activation and polarization. Classically polarized macrophages (M1 macrophages) are activated by LPS, IFN-ƴ and TNF-α. M1 cells have high microbicidal activity, immune-stimulatory functions and tumour cytotoxicity. Alternatively polarized macrophages (M2 macrophages) are involved with anti-inflammatory, wound repair and tumour promotion. M2 macrophages can be further divide into M2a, M2b, and M2c macrophages. IL-4 and IL-13 always activate macrophages to be M2a, while M2b are activated by immune complexes (ICs), TLRs, or IL-1ra. Finally, M2c macrophages are polarized by IL-10, TGF-β or glucocorticoids. All of the phenotypes express a series of different cytokines, chemokines and receptors

In general, monocytes/macrophages contribute to the modulation of the immune response can lead to autoimmunity. These cells are dynamic as to polarize phenotypes of pro-inflammatory and anti-inflammatory cytokines depending on the microenvironment, acting with different physiological functions [31]. Recently, macrophages are subdivided in two main phenotypes: the classically activated macrophages (M1 macrophages) promote tissue inflammation and activated macrophages (M2 macrophages), these last is classified into three subtypes according to their functions: host defence, tissue repair and immunoregulation [32] (Fig. 2).

Classically activated macrophages (M1 macrophages) intermediate host defence against a variety of bacteria, protozoa and viruses and participate in anti-tumour immunity, autoimmune diseases [33, 34]. Lipopolysaccharides (LPS) as certain bacterial products and cytokines such as interferon (IFN)-γ stimulate macrophages to M1 subset. As a result, M1 macrophages induce a strong pro-inflammatory phenotype with the production of cytokines (TNF-α, IL-6, IL-12 and IL-23) and chemokines (CCL-5, CXCL9, CXCL10 and CXCL5), promoting the recruitment of Th1 and Natural killer (NK) cells. In addition, it has been shown that M1 macrophages up-regulate the intracellular expression of a protein called suppressor of cytokine signalling 3 (SOCS3), which increases the production of reactive oxygen intermediates and nitrogen, the expression of MHC class II molecules and costimulatory molecules [35, 36]. In this perspective, M1 macrophages promote Th1 immune responses, but also contribute to the tissue destruction and tumoricidal activity [37]. Therefore, an over-activation can lead to tissue damage such as occurs in various inflammatory, autoimmune and chronic diseases, including Crohn’s disease, rheumatoid arthritis, diabetes, multiple sclerosis and autoimmune hepatitis [38–43].

Otherwise, alternatively activated macrophages (M2 macrophages) are involved with immune regulation, tissue remodelling, and elimination of parasites, tumour promotion and autoimmune diseases. The M2 macrophages can be divided into three differing subpopulations: M2a, M2b, and M2c macrophages [44–47]. They are clearly distinct, both functionally and biochemically [47]. In the context, macrophages contribute to the reparative extracellular matrix production, while regulators macrophages express high levels of costimulatory molecules (CD80 and CD86) and can present antigen to T-cells, although, there are differences among the subtypes M2 macrophages, usually macrophages repairers and regulators exhibit immunosuppressive activity [48].

The M2a macrophage is stimulated by IL-4/IL-13 through the binding of these cytokines to their receptors, which then activates STAT-6 signalling pathway. It also up regulates the histone demethylase JMJD3, by altering of chromatin modifications that induces expression of M2 gene and inhibits M1 gene during tissue repair and anti-inflammatory response [49, 50]. In addition, M2a macrophages up-regulated the Mrc1, resistin-like a (Retnla, Fizz1) and chitinase 3-like 3 (Chi3l3, Ym1) expression, suggesting that these expressions are selective markers of M2a macrophages [51].

M2b macrophages are polarized by combined immune complexes that contain toll-like receptor (TLR) and/or IL-1 receptor agonists [35, 52] and produce high levels of pro-inflammatory cytokines, including IL-1, IL-6 and TNF [53]. M2a macrophages also induce the influx of eosinophils, basophils, Th2 cells and regulatory T-cells by secreting CCL24, CCL17, CCL1 and CCR1 at the site of inflammation [54].

The M2c macrophages are induced by transforming growth factor (TGF)-β, glucocorticoids [37] or IL-10. In this context, IL-10 is secreted by dendritic cells, B cells, cytotoxic T-cells, T-cells, NK cells, mast cells, neutrophils, eosinophils, as well as monocytes/macrophages [55]. The activation mediated by IL-10 acts through a transmembrane receptor complex composed of IL-10R1 and IL-10R2. Thus, the IL-10/IL-10R1 interaction changes the cytokine conformation leading to its dimerization with IL-10R2, which activate Jak1/STAT3 signalling pathways [55].

These macrophages are engaged in a complex bidirectional interaction with neutrophils. They also can drive the development of the innate and acquired immune responses by complicated cross-talk with other cells, including natural killer and DC [56]. Various mechanisms underlying the neutrophils’ antimicrobial activity, such as phagocytosis, generation of reactive oxygen species (ROS), cytokines, chemokines, lipid mediators, degranulation of antimicrobials and enzymes [57]. In 2004, Brinkman et al. [17] demonstrated that neutrophils generate an extracellular chromatin fibre, called neutrophil extracellular traps (NETs), which disarm and kill extracellular bacteria [58]. The NETs are composed of DNA, histones, amphotericin HMGB1 (high-mobility group box 1), and globular structures that consist in components granules of neutrophils, such as neutrophil elastase (NE), myeloperoxidase (MPO), cathepsin G, proteinase 3, cationic bactericidal permeability increasing protein (BPI), calgranulin, α-defensins, lactoferrin, the peptide LL-37, pentraxin PTX3, matrix metalloproteinase-9 (MMP-9) and peptidoglycan recognition protein-S (PGRP-S) [59–61].

The mechanisms involved in the NETs formation are not fully elucidated. It was demonstrated that upon activation (by lipopolysaccharide, bacteria, fungi), neutrophils start a programmer that leads to their death by a mechanism distinct of apoptosis and necrosis, called NETosis [62]. For this, NE translocates to the nucleus, where it breaks, partially, specific histones, promoting chromatin decondensation [63], and the nuclei of neutrophils lose their shape, and heterochromatin homogenize [15]. The chromatin decondensation is made by the MPO enzyme, probably, due to the synthesis of hypochlorous acid [63]. Moreover, NETs formation depends on the ROS production by NADPH oxidase [22]. This ROS may alter several macromolecules, including DNA, proteins and lipids, making them more susceptible to attack by neutrophil enzymes [64]. In addition, the peptidyl arginine deiminase 4 (PAD4) induced catalyzes deamination of arginine residues in the histones intensifying chromatin decondensation [65, 66]. Finally, the nuclear envelope and the granule membranes disintegrate, allowing the mixing of NET components, then NETs are released as the cell membrane breaks [67].

**Fig. 3:**
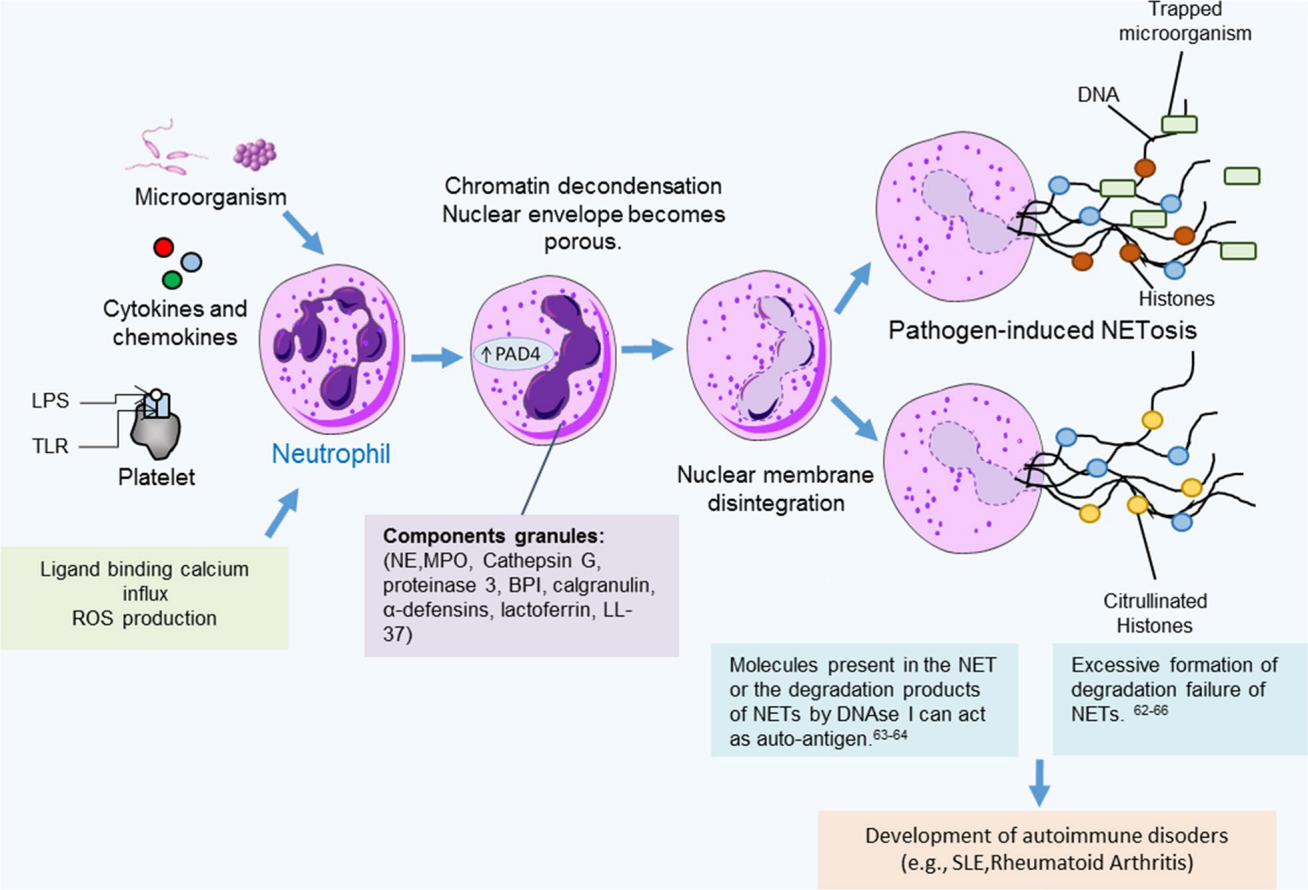
Neutrophil extracellular traps. Neutrophil extracellular traps (NETs) are composed of nuclear components (such as DNA and histones) and are decorated by proteins from primary granules (such as myeloperoxidase and neutrophil elastase), secondary granules (such as lactoferrin and pentraxin 3), and tertiary granules. NETs have been shown to trap microorganisms. Initially, the neutrophils become rounded with uniformly condensed chromatin, occurs because of PAD-4-mediated citrullination of histones, and then undergo nuclear envelope breakdown so DNA-containing vesicles eventually fuse with the plasma membrane, and NETs are released to trap microorganism. NETs can cause development of autoimmune disorders trough molecules present in the NET or the degradation products of NETs by DNAse I can act as auto-antigen or because excessive formation of degradation failure of NETs. Font: Adapted by [71], Mantovani et al. [72] and Phillipson and Kubes [245]

Initially, the formation of NETs was considered an innate immune response in response to infections. However, recent evidence suggests that these structures are involved in the pathogenesis of various diseases, including autoimmune disorders [62–65]. Regarding to autoimmunity, the molecules present in the NETs or the degradation products of NETs by DNAse I can act as auto-antigen [63, 64]. Several groups have proposed that the excessive formation or degradation failure of NETs that lead to the expression of a set of auto-antigens and danger-associated molecular patterns, are important factors in the development of autoimmune responses in predisposed individuals, as shown in Fig. 3 [62–66].

### The role of macrophages and neutrophils in the innate and adaptive immunity

Macrophages and neutrophils are qualified phagocytes that correspond to the first lines of defence against pathogens [68]. In general, the neutrophils were associated with acute inflammation, while monocyte/macrophages appear to be associated the chronic inflammation [69]. However, several studies have challenged these dogmas, showing that neutrophil is a key effector cell in the orchestration of the adaptive immunity in the resolution of chronic inflammatory response [70–72], while monocytes/macrophages are important in acute inflammation [73]. In this regards, neutrophils are the first leukocytes that are recruited rapidly into acute infection site, which after their activation, they kill or phagocytosis foreign bodies and release soluble mediators, as cytokines that induce the recruitment of monocytes to the inflammation site [69, 74, 75]. On this occasion, monocytes would then be recruited following neutrophils to the site of injury and differentiate into macrophages [1]. Certain studies suggested a predominance of monocytes/macrophages that substitutes the neutrophils. This propose a bimodal recruitment pattern that interchange neutrophils to monocytes [76]. However, currently there is evidence that chemoattractant, as MCP-1, produced in situ by tissue monocytes/macrophages rapidly and directly induces the monocytes influx regardless of the presence of infiltrating neutrophils at inflammation site [1]. Thus, neutrophils and monocytes found into the inflammatory site can participate in both the innate and adaptive immune response, displaying several functions described in Table 1.

**Table 1:** General functions of macrophages and neutrophils in innate and adaptive immunity

	Monocyte/macrophage	Neutrophil
Innate immunity	Opsonic recognition [229] Production of pro-inflammatory and anti-inflammatory cytokines [230] Release GCSF and GM-CSF [231] Excessive release of toxic species (NO, superoxide and MMP) [232] Antigen processing, and presentation [233]	Production of prostaglandins, leukotrienes and proteases [234] Release MIP-1α and MIP-1β [235] Release cytokines, such as IFN-γ, IL-8 and TNF-α [71] Secretion of antimicrobial molecules [236] Phagocytosis [237] Release lytic enzymes and producer active oxygen intermediates [71] Release neutrophil extracellular traps (NETs) [71]
Adaptive immunity	Secretion of hydrolytic enzymes [238] Cleavage of C3 [239] Induce neovascularization and contribute to angiogenesis and lymphangiogenesis [240] Modulate the osteoclastogenesis [241] Efferocytosis [242] May induce Th1 cells differentiation [78] Control the effector T-cell homeostasis, promoting the T-cell priming and also may induce Th17 cell differentiation [81]	Release IL-17 [243] Release NETs [71] Promote the maturation of human monocyte-derived DC [88] Chemotaxis of Th1 and Th17 [59, 60] Differentiation of naïve CD8+ T-cells [244]

IFN-γ: interferon gamma; TNF-α: tumour necrosis factor-α; IL-1β: interleukin-β; GCSF: Granulocyte colony-stimulating factor; GM-CSF: Granulocyte macrophage-colony stimulating factor; MIP-1α: macrophage inflammatory protein-1α; NETs: Neutrophil extracellular traps

In this context, monocytes ∕ macrophages play a central role in both adaptive and innate immunity, due these cells play dual role in tissue injury, either injury-inducing or repair-promoting [73]. Thus, macrophages constitute an important class of antigen-presenting cells (APC) that activate adaptive immunity, as well act in phagocytosis, antigen processing and presentation, leading to the activation of T and B cells. These cells also secrete pro-inflammatory, anti-inflammatory, angiogenic, fibrogenic or mitogenic cytokines. These processes cooperate with others immune and progenitor cells to control the initiation, resolution, and repair tissue damage during chronic inflammation [73, 77]. In summary, this cell has a large repertoire of well-characterized abilities and functions both in innate and adaptive immunity, including regulation of inflammatory responses, stimulation of T and B cells, may promote Th1 cells differentiation, tissues homeostasis and development, repair of damaged tissue, rejection of a xenograft, induce angiogenesis and lymphangiogenesis, modulation of osteoclastogenesis, as well as macrophages are key component in elimination of pathogens and removal of dying cells by efferocytosis [5, 78–80]. In addition, resident tissue macrophages also are patrollers in epithelial tissues, ensuring the entry and colonization sites for pathogens in order to prevent the invasion of these infectious agents [80]. An important example is the alveolar macrophages, which keep the lung surface under surveillance-inhaled pathogens [5]. In adaptive immunity, recent studies reported that macrophages may control the effector T-cell homeostasis, promoting the T-cell priming and also inducing Th17 cell differentiation [81]. Also, macrophages modulate cytokine release and T-cell activation, resulting in neuropathic pain [82].

After injury, with or without infection, neutrophils recruited into tissue have an anti-infectious and pro-inflammatory function, due to their ability to phagocyte and to produce powerful components for the host, i.e., ROS, such as O_2_^−^, H_2_O_2_, HOCl, and ^·^OH; antimicrobial peptides, proteolytic enzymes as serine proteases and metalloproteinase; and release NETs, leading to tissue injury [83–85]. However, recruited neutrophils are mostly removed by DC and macrophages at the site of inflammation by a process of cell corpse removal called efferocytosis [86].

Even though neutrophils are the hallmark effector cells of acute inflammation, these cells also contribute to chronic inflammation and adaptive immune responses, such as cytokines (as IL-17) secreted from neutrophils regulate the immunity by inducing the expression of pro-inflammatory factors (such as cytokines, chemokines and MMPs) from mesenchymal and myeloid cells, leading to the perpetuation of the recruitment and activation of additional neutrophils in chronic inflammation [87]. In addition, neutrophils directly interact with macrophages, DC, and lymphocyte subsets and modulate their effector functions; as well as promote the maturation of human monocyte-derived DC (moDC) [88]. They interact with DC leading to activation of NK cells and communicate with the B and T-cells and NK cells, as well as cross-interact with NK cells that produce IFN-γ, which promotes survival and activation of the neutrophil [87, 89, 90]. Thus, activated neutrophils secrete chemoattractant, such as CCL2, and CXCL10 or CCL2 and CCL20, which attract and recruit Th1 and Th17 cells, respectively [91, 92]. Neutrophils may express MHC II class and act as APC, thus it migrates to the lymph node attracted to CCR7 [93–95]. Furthermore, the neutrophils can also present exogenous antigens via MHC I class, promoting the differentiation of naive CD8+ T-cells into cytotoxic T-cells [95]. On the other hand, neutrophils can also compete with the professional APC and thus inhibits the T CD4+ response [95, 96].

Due the ability of neutrophils and macrophages to initiate and control the immune responses against invading pathogens as well as against self-proteins derived peptides, thereby they play an important role in the pathogenesis of autoimmune disorders, such as autoimmune diabetes, rheumatoid arthritis and Systemic Lupus Erythematosus (SLE). However, the nature of the contribution of these cells in autoimmune disorders is not yet fully elucidated, but there are several important factors affecting on the immunogenicity of these cells, including the type and dose of antigen, the microenvironment of the cell-antigen encounter, the number, the subset, and phenotype of these cells, which can prevent or induce autoimmune responses. In this regard, Orme and Moah [6] reported important alterations of these cells during the establishment of SLE such as: (1) enhanced apoptosis; (2) enhanced chemotaxis due the overexpression of MCP-1, MIP-1α, CCL5, CXCR4, CXCL12; (3) impaired phagocytosis related to high levels of the complement receptor CR3 (CD11b/ITGAM) and of FCγR1; (4) impaired immune—complex clearance; (5) and impaired superoxide production.

**Fig. 4:**
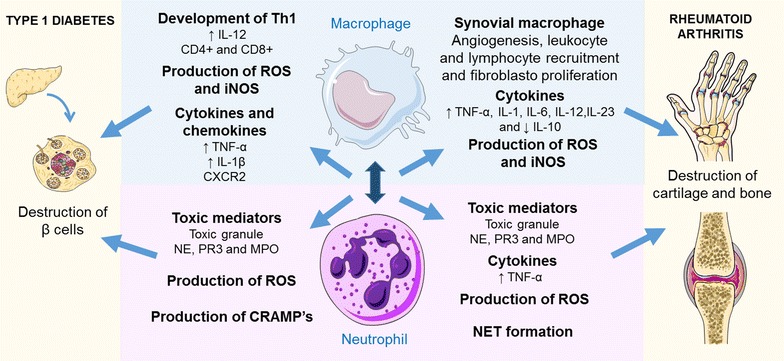
Major mechanism of action of neutrophils and macrophages in type 1 diabetes and rheumatoid arthritis

Overall, most studies indicate that adaptive immunity alone is involved in autoimmune disease. However, other studies have suggested crosstalk between cellular and humoral components involved in the innate and adaptive immune systems, which indicates that the loss of the immune homeostasis between two important pro-inflammatory and anti-inflammatory effectors may trigger the effector phase of autoimmune diseases, as shown in Fig. 4 [97, 98].

### Crosstalk between neutrophils/macrophages in autoimmune type 1 diabetes

Autoimmune type 1 diabetes mellitus (T1DM) is a serious chronic autoimmune disease characterized by an absolute insulin deficiency, resulting from an depletion of insulin-secreting β cells located in pancreatic Langerhans’ islets mediated by autoantigen-specific CD4(+) and CD8(+) T-lymphocytes promoting further inflammation in this tissue [99–101]. In this regard, innate immune cells such as γδT-cells, NKT, macrophages and neutrophils play essential roles in the pathogenesis of T1DM [102]. These cells produce cytokines that promote β-cell apoptosis and increase infiltration of islet specific T-cells, then this T-cells attack and destroy β-cells, resulting in an insulin deficiency [102].

A lack of insulin results in an inability to regulate blood glucose effectively, leading to chronic hyperglycaemia. Therefore, T1DM may provide a state of hypoinsulinemia and hyperglycaemia, and causes serious health complications including ketoacidosis, kidney failure, heart disease, stroke, and blindness [103]. In diabetes cases, approximately 5–10% of diabetics have T1DM [104], which can be classified into two categories: Type 1A diabetes mellitus, resultant of an autoimmune response destroying the β-cells, while type 1B is an idiopathic destruction of the β-cells [104]. Type 1A diabetes mellitus has a predominantly genetic origin, there are at least 15 loci associated with T1DM, mainly polymorphic genes, such as the human leukocyte antigen (HLA) loci, interleukin 2 receptor alpha (IL2R2), insulin-variable number tandem repeat (VNTR), vitamin D receptor (VDR), cytotoxic T-lymphocyte-associated protein (CTLA)-4 genes [102, 105, 106]. For example, HLA class II haplotypes either DR3DQ2 or DR4DQ8 are express in more than 90% of patients with T1DM [107]. In general, the triggers that initiate the immune-mediated destruction of the β-cells include viruses (Coxsackie B viruses, rubella viruses, cytomegaloviruses and retroviruses), dietary factors (cow’s milk, plant proteins, nitrates, wheat gluten) and other environmental factors (e.g., caesarean delivery, early childhood diet, vaccines and use of antibiotics), as well as gut microbes [102, 108].

In addition, hyperglycaemia may be a trigger to inflammatory response in T1DM, because it may increase the levels of circulating C-reactive protein (CRP), free fatty acids, CXCL-8, and pro-inflammatory cytokines, such as IL-6, IL-1β and TNF-α [109–111]. Hyperglycaemic conditions can also facilitate the activation of human T-lymphocytes [111, 112]. Therefore, the innate immunity plays a critical role in T1DM, because various cells, including macrophages, dendritic cells, NK cells and neutrophils, may recognize pathogens and foreign molecules without having been previously exposed to them and without generating long-term immune response [102].

In acute inflammation, macrophages, mainly the M1 macrophages, are first cells accumulate at one side of the pancreatic islet at an early stage (2–3 weeks of age), along with neutrophils, dendritic cells (DCs) and B lymphocytes [113]. In this regard, M1 macrophages can trigger an inflammation and initiate pancreatic β-cell death and insulitis during type 1 diabetes [114, 115], as well as it contributes to prolongation of inflammation instead of its resolution [114]. The M1 macrophages are pro-inflammatory cells that responds to intracellular pathogens producers of TNF-α and interleukins (IL-1β, IL-6, IL-8, IL-12, and IL-23), express costimulatory molecules important in T-cell activation (MHC, CD40, CD86), and produce proteases (leucocyte elastase, and matrix metalloproteinases), such as NO through the expression of iNOS and ROS [116]. Therefore, ROS can induce apoptosis or necrosis of β cells by activating the caspase pathway and inducing excessive cell stress, respectively, because this cell is very sensitive to free radicals and has low free radical scavenging activity [116, 117].

During the chronic inflammation, the IL-12 from M1 macrophages can contribute to destruction of β cells in T1D by directing the development of Th1 cells, with the involvement of CD4+ and CD8+ lymphocytes [118, 119]. Furthermore, activated M1 macrophages have an enhanced ability to present antigen and then stimulate the activation of CD8+ T-lymphocytes, which are directly involved in the destruction of pancreatic β-cells [115, 116, 120]. In animal model, certain studies also pointed to the evidence for initial involvement of macrophage in development of insulitis in non-obese diabetic (NOD) mice [121, 122]. Other study showed in NOD mice that macrophages from pancreas produced more IL 1β than peritoneal macrophages [123]. This cytokine production mediates the damage outcome of macrophages on β-cells [124].

However, both macrophages M1 and M2 phenotypes play central role in diabetes.

Similarly, the M2 macrophages phenotypes have also been reported for NOD mice [120, 125] and in human T1D [126, 127]. However, M2 macrophages have distinct functions of M1 macrophages, because the M2 macrophages decrease hyperglycaemia, insulitis and inflammation in the pancreas [116]. In this regard, the adoptive transfer of immunomodulatory M2 macrophages prevents type 1 diabetes in NOD mice [128]. In short, the characterization of subpopulations of macrophages in diabetes may be useful in designing new strategies to T1DM understanding. Several evidence reported that M2 macrophages secretes anti-inflammatory cytokines IL-4/IL-13 and expresses high levels of PD-1 ligands (Program-Death 1), PDL-1 and PDL-2, thereby this mechanism may inhibit the proliferative response of activated T-cells in T1DM [116].

Regarding to neutrophils, Battaglia [100] highlighted that these cells are crucial in the initiation and perpetuation of T1DM, during acute and chronic inflammation, by two proposed mechanisms: (1) one is due the production of pro-inflammatory cytokines, as IL-17, which promote granulopoiesis and consequent neutrophil proliferation and accumulation, leading to prejudice pancreatic cells; and (2) another involves the unintended entrance of bacteria on pancreas that release toxins, leading to the production of IL-6 and IL-8 that attract neutrophils. Therefore, immune cells in pancreas, especially macrophages and neutrophils are responsible to the initiation and perpetuation of T1DM. Other study reported by Huang et al. [56], showed that several indications suggest the involvement of neutrophils in the initiation and perpetuation of autoimmune diabetes, such as an increased numbers of neutrophils in blood of type 1 diabetes patients, activation and recruitment of neutrophils to pancreatic islets in T1DM patients, liberation of neutrophil toxic mediators and the antineutrophil treatments were able to reduce the insulitis and autoimmune diabetes.

In this context, in diabetic ketoacidosis patients (DKA) have been found monocytosis and neutrophilia in differential white blood cell count, consistent with previous reports of increased circulation of leukocytes during DKA [129]. Although, there are controversies regarding the exact roles of neutrophils, this cell was found in the pancreas of patients with T1DM, both at the onset and at later stages of the autoimmune process [130]. Some studies have shown an increased adherence of neutrophils to the cerebrovascular endothelium in diabetic ketoacidosis patients [109, 131]. In diabetic ketoacidosis patients were found activated neutrophil with high release of toxic granule content in serum, as elastase, proteinase-3 and myeloperoxidase [132]. Also, patients with T1DM had markedly elevated levels of NE and proteinase 3 (PR3), which are serine proteases stored in neutrophil primary granules [133]. On the other hand, other studies reported that neutrophils from insulin-dependent diabetics display diminished levels of oxygen radicals (${\text{O}}_{2}^{ - }$) and activation of phospholipase D in response to FMLP, which was attributed to the high glucose concentration in these patients [134]. Also, certain studies have shown that circulating neutrophil number was decreased in patients with T1DM, which may associated with abnormal neutrophil yield and maturation, peripheral consumption or damage, and tissue detainment [130, 135].

In animal studies, Alba-Loureiro et al. [136] showed that neutrophils from diabetic rats exhibited decreased phagocytosis and phorbol myristate acetate (PMA)-stimulated H_2_O_2_ production. Similarly, Nurun Nabi et al. [137] reported that neutrophils from diabetic rats were more activated at their basal level, though it exhibited lower morphological polarization in response to FMLP. These authors also demonstrated that in increased plasma glucose level can reduce the phagocytosis of neutrophils from diabetic rats [137]. In addition, Diana et al. [138] observed the occurrence of neutrophils inside the pancreatic infiltrate of NOD mice, but not in non-autoimmune C57BL/6 and BALB/c mice [138]. Another study in NOD mice showed that one of the chemokine receptor that regulates the trafficking of neutrophils to pancreas is the CXCR2, which ligands are produced by pro-IL-1β secreted by pancreatic macrophages [123]. Thus, the role of neutrophils in T1D started to be elucidated. The presence of neutrophils that secrets cathelicidin-related antimicrobial peptide (CRAMP) was observed in NOD mice histological analysis [138]. In others autoimmune diseases, CRAMP’s stimulates the plasmacytoid dendritic cells (pDCs) to produce IFN-α. Therefore, immune cells in pancreas, especially macrophages and neutrophils are responsible to the initiation of T1D.

Together, the current findings indicate that high levels of chemokines, such as CXCL1/KC (or human IL-8) and MCP-1/CCL2, are secreted into the pancreas during the T1DM. These chemokines play a key role in the recruitment of neutrophil and macrophages/monocytes, respectively, from the blood vessels to the pancreatic islets [139–141]. In this regard, as shown above, human and animal studies showed that macrophages and neutrophils were found to infiltrate islet of young NOD mice and T1DM patients, and the inhibition of influx these cells into the pancreas, by depletion or inhibiting their adhesion molecules, prevents the development of insulitis and inflammation in T1DM [114, 130, 142–144]. These facts, taken together, suggest that macrophages are cells important in stimulation of the influx and activation of cells, including T-cells, which lead to destruction of the β cells. After started the inflammatory process, other cells are recruited to the pancreas, such as neutrophils, which at the beginning of the pathology are involved in the propagation of tissue damage through the release of toxic chemicals. Thus, these findings support the evidence for a crosstalk between macrophages and neutrophils that participate in closely in the inflammatory response and progression of the initial pancreatic damage in T1DM.

### Crosstalk between neutrophils/macrophages in rheumatoid arthritis

Rheumatoid arthritis (RA) is an inflammatory autoimmune disease characterized as a chronic inflammation within the synovial tissues in multiple joints (synovitis), leading to progressive and erosive destruction of cartilage and joints, and the underlying bones [145]. The pannus formation and synovial hyperplasia are the main features of RA, due an abundant cellular infiltrates of several cell types (neutrophils, macrophages, fibroblasts, T-cells, and dendritic cells) in the synovial [146, 147]. Furthermore, aetiology and pathogenesis of RA have not been completely elucidated, but it is widely accepted that is a multifactorial disease associated with genetic factors (HLA genes, as HLA-DRw4 and HLA-DRB1, PTPN22 (encoding tyrosine-protein phosphatase non-receptor type 22) gene, protein-arginine deiminase type 4 (PADI4) gene and cytotoxic T-lymphocyte protein 4 (CTLA-4) gene), environmental (e.g., cigarette smoke), gender and age [148–150]. In this context, certain studies report potential roles for sex hormones and sex chromosomes in induction of RA [151, 152].

Furthermore, environmental factors may lead molecular changes to host proteins, followed by breakdown of immune tolerance to self, thereby increase the risk of developing RA in individuals with genetic risk factors [153]. Certain environmental exposures induce peptidyl arginine deiminase (PAD) enzyme activation and consequently protein citrullination [154, 155] that in turn lead to development of autoantibodies to citrullinated protein antigens (ACPAs), which are detected in RA patients serum [156]. ACPAs are directed against different citrullinated antigens, including fibrinogen, fibronectin, α-enolase, collagen type II, histones. Furthermore, these antibodies are detected years before the onset clinical arthritis and are associated with joint radiographic damage and extra-articular manifestations [157]. In the RA, there are many cell types that play a role in the pathogenesis, such as fibroblasts, T-lymphocytes, B lymphocytes, neutrophils and macrophages [158]. It is known that there is an imbalance between pro and anti-inflammatory cytokines that can be a key mechanism underlying disease progression in collagen-induced arthritis (CIA) mice model as well as in human RA [159].

In this context, neutrophils are the first effector cells at the site of inflammation, mainly in the initiation of various pathologies including chronic inflammatory diseases. Thereby, these cells play a key role in the tissue damage and facilitate the inflammatory process in RA, leading to joint destruction in arthritis models [160, 161]. In animal models studies, the neutrophil depletion with antibodies blocked or reversed the joint swelling and joint inflammation in arthritis [162]. In addition, neutrophils are the most abundant both in the synovial fluid (SF) and pannus of patients with active RA. Chemoattractants such as GCSF and IL-8 stimulate neutrophil migration from the peripheral blood to the joint in RA [163], as well as promotes neutrophil trafficking into inflamed joints [164]. In this context, some studies showed that neutrophils from RA patients are functionally different from those of healthy individuals, because they have delayed apoptosis and are more susceptible to stimulation via TLRs and receptors for complement fragments, growth factors, cytokines and immune complexes [165–168].

Once activated, in acute inflammation of RA, neutrophils release high concentrations of oxidants and cytotoxic products, such as ROS, cytokine (such as TNF-α) and granules containing proteases, phospholipases, defensins and myeloperoxidase, in the synovial fluid or directly onto the surface of articular cartilage [166, 167, 169]. Moreover, Wright et al. [156] summarized the relevant role of certain neutrophil granule enzymes found in high concentrations in RA synovial fluid, such as contributes to the destruction of the articular cartilage and tissue (e.g., elastase, gelatinase and collagenase), promotes inflammation and activate cytokines/receptors (e.g., elastase, cathepsin G, proteinase 3 and lactoferrin), as well as inhibits resolution of chronic inflammation and cartilage repair (e.g., myeloperoxidase and gelatinase-associated lipocalin). In addition, other effects also were described, including inhibit chondrocyte apoptosis (e.g., lactoferrin), and regulating migration, invasion and proliferation of synoviocytes (e.g., cathepsin and matrix metalloproteinases) [170–176].

In chronic inflammation of RA, several studies have linked neutrophil functions to Th17 cells. These cells are producers of IL-17 that is a potent pro-inflammatory mediator implicated in the pathogenesis of RA, involved in induction of tissue inflammation by stimulating the recruitment of neutrophils. In the RA joint, IL-17 also activates fibro-blast-like synoviocytes, macrophages, and osteoblasts [91, 92, 177]. Thus, these activated cells in the joint produce potent neutrophil chemoattractants, as IL-8 and TNF-α, which in combination with IL-17 stimulate synovial endothelial cells to produce more neutrophil chemoattractants [91, 92, 177]. Thereby, neutrophils found in the RA joint help sustain Th17 cells through the secretion of Th17 chemokines, as CCL20 and CCL2 [91, 92]. In addition, synovial fluid and peripheral blood neutrophils from patients with osteoarthritis are more likely to form NETs than in neutrophils from healthy controls [177]. In this regard, NETs also are source of citrullinated protein, as such histone and vimentin, because during NETosis occurs hypercitrullination of histones induced by the enzyme peptidyl arginine deiminase type 4 (PAD4) [178]. The ACPAs in the serum of patients with RA react with histone H4 in the NETs [179] and are correlated with disease activity and the severity of joint destruction [153]. Indeed, RA serum, anti-antibodies, as well as inflammatory cytokines IL17A and TNF, induce NETosis in RA neutrophils [180]. Overexpression of IL-17A in healthy mouse knee joints induced rheumatoid arthritis (RA)-like pathology with features including joint inflammation, focal bone erosion and cartilage damage [181]. In fact, NETosis is enhanced in circulating and synovial fluid neutrophils from patients with RA and it was observed NETs infiltrated in RA synovial tissue, rheumatoid nodules, and skin, mainly in patients with high levels of ACPA. In addition, NETs enhance inflammatory responses in RA synovial fibroblasts by stimulating production of IL-8 that may further enhance NETosis [182], citrullinated autoantigen exposure, and promote autoantibody generation amplifying mechanism of joint damage [183]. In conclusion, NETs externalize various immunostimulatory molecules and citrullinated auto 1 antigens that, in predisposed individuals might account for persistent generation of ACPA.

Therefore, in summary, there is strong evidence that the neutrophils have multiple functions in regulating acute and chronic inflammation in RA, such as: (1) produce mediators, such as IL-10, IL-1 receptor antagonist (IL-1ra) and TGF-β, with play an important anti-inflammatory role during both acute and chronic microbial infections or that contribute to resolution of inflammation; (2) secrete pro-inflammatory and anti-inflammatory cytokines and chemokines that regulate the function of immune cells (e.g., active macrophages, promote activation, proliferation and differentiation of T-lymphocytes, recruitment and maturation of dendritic cells) [156, 184, 185]; (3) Human neutrophils express MHC class II (HLA-DR) and co-stimulatory molecules (e.g., CD80 and CD86) that stimulate superantigen-dependent T-lymphocytes activation, as well promote differentiation of the Th1 and Th17 effector T-cell subsets [94, 186–188]; (4) Activated neutrophils express the B cell–activating cytokine (BLyS or BAFF) and the interaction between neutrophils and marginal zone B cells mediate T-cell–independent antibody responses through BAFF and a proliferation-inducing ligand (APRIL) [189–193]; (5) Neutrophils play a role in regulating the homeostasis; terminal differentiation and functional responsiveness of NK cells in human and mice, as well as neutrophil-derived mediators modulate NK cell effector functions, possibly in close relationship with dendritic cells [88, 156, 193]. Other functions also were reported, as synovial fluid neutrophils of patients with exacerbation of RA strongly express receptor activator of nuclear factor kappa-B ligand (RANKL) that activate the osteoclastogenesis [166, 194], while peripheral blood neutrophils from both RA patients express B lymphocyte stimulator (BLyS or BAFF), which is implicated in regulation of B cell-dependent autoimmunity [195].

Macrophages are one of the resident cell types in synovial tissue, along with fibroblasts [196]. In the context, once activated macrophages have a critical role in RA in chronic inflammatory arthritis and these cells have high plasticity, differentiating into different phenotypes, which can secrete either pro-inflammatory (M1 macrophages) or anti-inflammatory (M2 macrophages) cytokines [46, 197]. Both macrophages types are important to mediate matrix destruction or deposition, as well as help to resolution of inflammation [44, 196, 198]. During the last years, several studies have found greater numbers of M1 macrophages in synovial membranes of patients with RA and its depletion using specific antibodies can prevent their presence in the pannus and thus attenuate inflammation [199–201]. In addition, monocytes/macrophages are also associated with pathological bone erosion on RA, because these cells differentiate into osteoclasts, specialized cells in bone resorption [202].

Overall, synovial macrophages may stimulate the angiogenesis, leukocyte and lymphocyte recruitment, fibroblast proliferation, and protease secretion leading to eventual joint destruction [203–205]. M1 macrophages have around 30–40% of the cellular content, and secrete pro-inflammatory cytokines (TNFα, IL-1, IL-6, IL-12, IL-23, and low levels of IL-10) and enzymes involved in driving the acute inflammatory response and joint destruction [196]. In addition, high levels of pro-inflammatory cytokines and chemokines also contribute to the cartilage and bone destruction, and in the pannus formation in RA [119]. In this context, chemokines released by M1 macrophages promote the recruitment of leukocytes to the inflamed joint, which produce more pro-inflammatory mediators as IL-1, TNF-α IL-6 and matrix metalloproteinase leading a destructive potential synovial and bone, mainly in osteoarthritis [147]. Therefore, the IL-1 and TNF-α are the most abundant cytokines in the inflamed synovium, leading to synovial inflammation and activate chondrocytes and synovial fibroblasts. Thus, these cells produce IL-6, IL-8, and leukocyte inhibitory factor, as well as stimulate protease and prostaglandin production in synovia [206–208]. In addition, IL-1 and TNF-α also induce the expression of other cytokines (e.g., IFNγ), cell-adhesion molecules, chemokines and chemokine receptors, antigenic factors and lipid mediators and inducible nitric oxide synthase (iNOS) in the inflammatory site [41, 196]. The TNFα and other molecules also induce the histone acetyltransferase (HAT) activity in macrophages, leading the acetylation of histones and subsequent modulation of transcriptional factors [196]. The IFNγ is another cytokine highly expressed in RA synovial tissue and its elevated levels correlates with RA severity [209]. In this context, pro-inflammatory cytokines, like TNFα, can upregulate the production of INFγ that increase the response of M1 macrophages, while IL-10, anti-inflammatory cytokines downregulate this effect [210, 211]. Therefore, Wallet et al. [211] showed that IFNγ-primed activated macrophages produced elevated levels of TNFα and other TH1 cytokines, as IL-12p70, but not of regulatory cytokine, as IL-10. Regarding to IL-10, Ji et al. [212] reported that in RA occurs a suppression of IL-10 signal transduction by blocking the FCγ receptor ligation induced by the combination of IFN and immune complexes found in RA, thereby a dysregulation of IL-10 signalling by these factors contribute to pathogenesis of RA.

On the other hand, M2 macrophages, during chronic inflammation, produce anti-inflammatory cytokines that is associated with tissue remodelling and immunoregulatory functions, improving the pathogenesis of RA [196, 212]. Thereby, the inhibition of macrophages should be a strategy of inhibiting inflammation and bone erosion in arthritis. Thus, the switch from a pro-inflammatory phenotype (M1) to an anti-inflammatory state (M2) can contribute to ameliorate the disease. Their plasticity makes them al target for the treatment of inflammation, especially arthritis. In this regard, certain studies reported that M2 macrophages profile is involved in spondyloarthritis (SpA) pathogenesis compared to RA patients, while M1 mediators correlate with joint inflammation in RA [213]. Therefore, Ambarus et al. [200] reported increased number of CD163+ macrophage (M2) phenotype in spondyloarthritis (SpA) synovitis, but not in RA, as well as these authors showed lower levels of M1-derived cytokines (pro-inflammatory) in SpA compared with RA synovial fluid. Therefore, certain studies have used M2 polarizing cytokines like IL-10 as therapy target, showing that IL-10-treated animals exhibited suppression of the development and progression of joint inflammation, even in established disease [214–216].

Moreover, other studies reported a possible key for macrophages in RA development in part by successful treatment of RA by anti-TNF antibodies [217–219]. The block TNF-α resulted in the inhibition of IL-1b, IL-6 and IL-8 production [220–222]. Also, in RA, high levels of IL-17 and its receptor are found in RA synovial fluid and tissues [223]. In this context, researchers have suggested that the IL-23/IL-17 pathway, rather than the IL-12–IFN-γ axis, is essential to promoting the development of CIA [224]. Indeed, the IL-10 inhibits IL-17 and RORγt expression in macrophages and suppresses macrophages toward the pro-inflammatory M1 phenotype, which is important for the role of IL-10 in mediating the pathogenesis of CIA [225, 226]. In addition, M1 macrophages have the ability to drive the CD4+ T helper cell polarization; thereby it can trigger B-cell and production of immunoglobulins and rheumatoid factor in synovia [227, 228].

These findings reveal a crosstalk between neutrophils and macrophages, which may result in a cascade of reactions leading to the destruction of host tissues, mainly during inflammation chronic, associated with RA. In short, after the initiating event, neutrophils are one of the first cells attracted to the site of inflammation, which together with synovial macrophages or recruited release ROS, cytokines, chemotactic factors, and granules enzymes, such as collagenases, proteinases, and elastases, accentuating the inflammatory response that result in cell damage in the synovial fluid of the joints during the RA. Neutrophil death or degranulation or formation of NETs may result in the release of soluble products into the extracellular environment where are scavenged by macrophages. In addition, neutrophils and macrophages participate in the regulation and activation of other cell types such as T and B lymphocytes, dendritic cells, NK cells. Thus, our data suggest that mechanisms involving these cells and their mediators can be important for understanding both the pathology and possible therapeutic interventions for RA.

## Concluding remarks and future directions

As described in this review, neutrophils and macrophages share the same origin and also have a number of common functions (e.g., pathogens phagocytosis, similar kinetic behaviour during the process of inflammation and immunomodulatory properties). Furthermore, it is well elucidated that neutrophils and macrophages are important cells of both the innate and acquired immune response to fight infectious agents. However, products released by these cells during the inflammatory process can also recruit and/or activate other cell types such as epithelial cells, endothelial cells, platelets, T and B lymphocytes, NK cells, among others. Thus, these factors set contribute effectively to the development of several autoimmune diseases such as rheumatoid arthritis and diabetes and others, as well as discussed in this review.

**Fig. 5:**
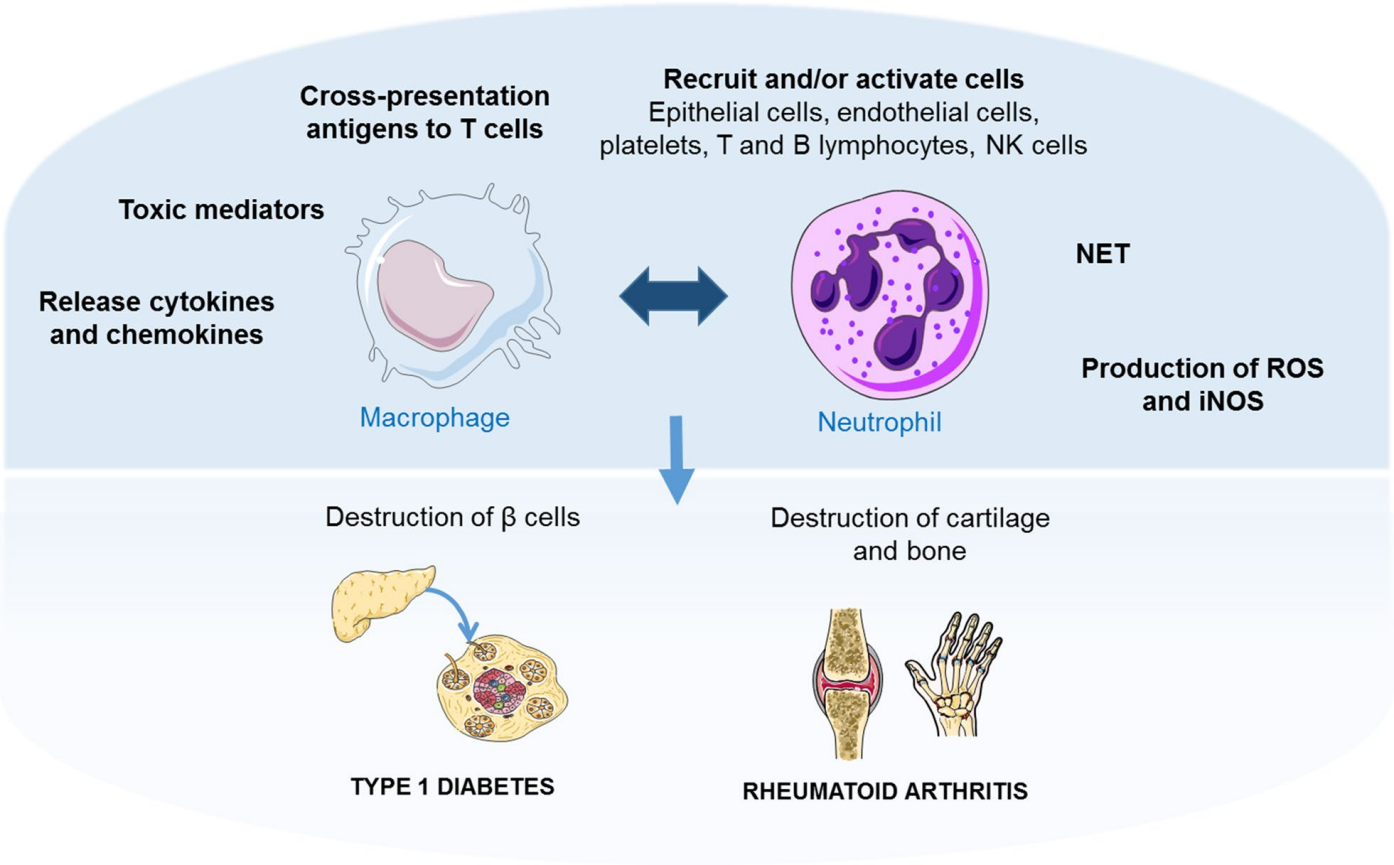
A simplified schematic of the crosstalk between neutrophils/macrophages in rheumatoid arthritis

Nonetheless, neutrophils have an important role recruiting and activating macrophages to the site of infection or acute inflammation. Therefore, the interaction of neutrophils and macrophages is a key event in innate immune response in the autoimmune diseases. In summary, during the inflammatory process, macrophages migrate at the same time or after the influx of neutrophils into the inflamed tissue, taking a direct or indirect interaction between these cells. As previously reported, macrophages of profile M1 secrete TNF-α, and along with neutrophils contribute to local inflammation, while M2 secrete IL-4 and IL-10 that modulate the inflammation, improving the tissue damage. Moreover, M1 macrophages are generated starting from cellular immune responses, and vital to the defence of the host. However, an exaggerated activation of these cells can lead to tissue damage as it is seen in many chronic inflammatory and autoimmune diseases, including type 1 diabetes, rheumatoid arthritis and others. In this regards, neutrophils can release NETs, molecules that are associated the induction of several autoimmune diseases, due they acted as auto-antigen, as well as the excessive formation or degradation failure of these NETs can lead the auto-antigens expression and danger-associated molecular patterns. Furthermore, neutrophils can migrate to lymph nodes and to regulate the functions of macrophages/DC, leading to cross-presentation antigens to T-cells. In addition, neutrophils can express MHC class II and co-stimulatory molecules that directly to activate superantigen-dependent T-lymphocytes and help the differentiation of the Th1and Th17 effector T-cell subsets. Also, activated neutrophils may express the BLyS or BAFF that mediate the T-cell–independent antibody responses of these cytokines, and then cooperating to activation of the adaptive immune responses. Thereby, many studies corroborate with the knowledge that neutrophils and macrophages, during infection and autoimmune diseases, play an important role in regulating of both T and B cells and activating other immune mechanisms, as shown in Fig. 5.

**Fig. 6:**
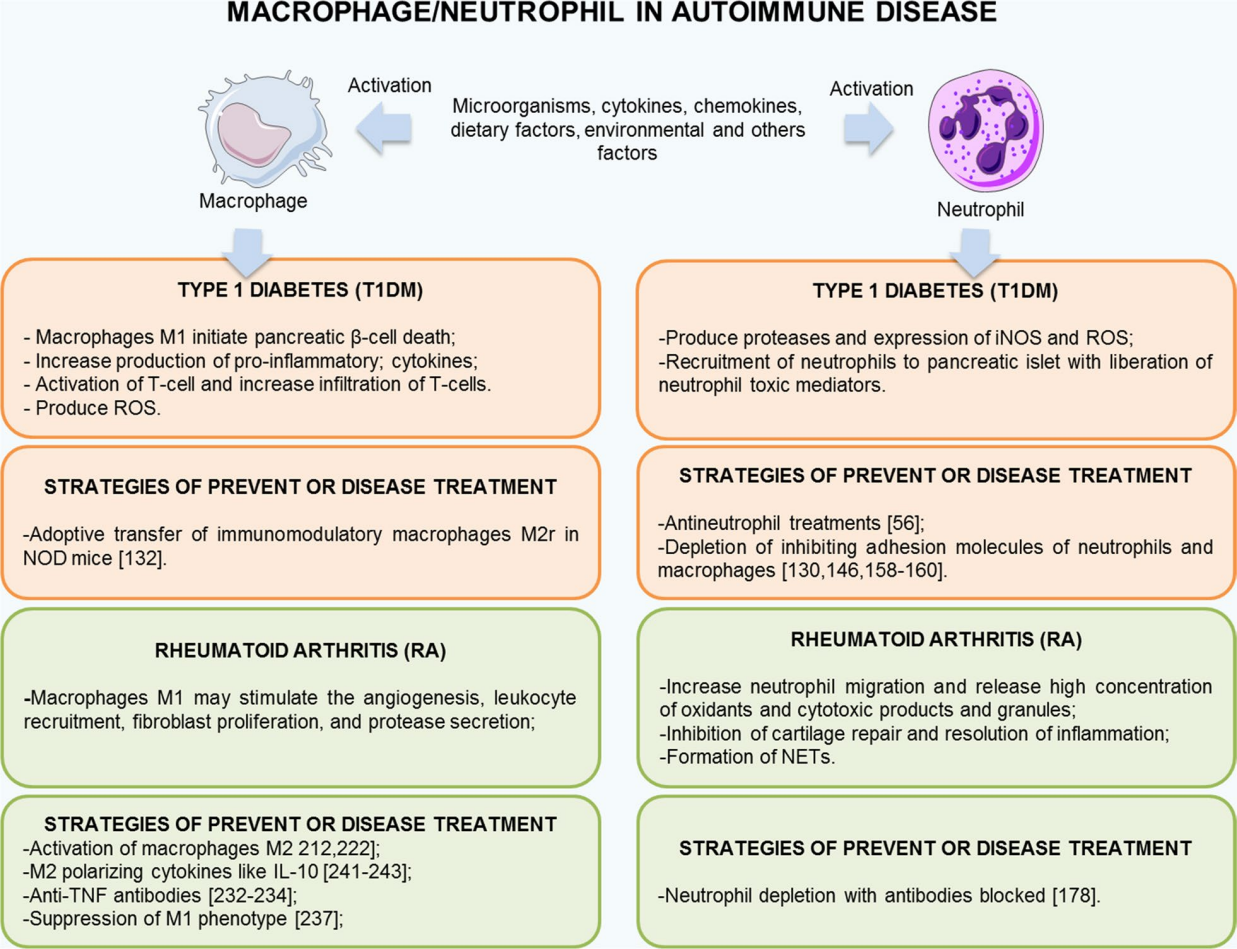
A simplified schematic of the role of macrophage/neutrophil in autoimmune diseases type 1 diabetes and rheumatoid arthritis

However, the major limitation currently facing these studies is the lack of studies that relate the functions in sets of cells involved in the pathogenesis of autoimmune diseases. This is likely due to the complexity of measuring the interactions among neutrophils and macrophages and their mediators that are involved in these disorders. Most studies focus on these characteristics of a targeted cell or molecule under in situ conditions. Important questions thus persist regarding, for instance, how to relate the plasticity and diversity of macrophages and neutrophils, which are quite important in mediating the innate and adaptive immune responses leading to tissue damage in type 1 diabetes and rheumatoid arthritis, as well as how these factors may help to develop multiple interventions that address the inflammatory responses and tolerogenic roles of these cells in these diseases, as shown in Fig. 6. However, future studies might examine whether therapeutic interventions that simultaneously modulate certain roles of macrophages and neutrophils can also improve of symptoms and prevent organ damage in autoimmune diseases. These findings remain to be clarified in order to elucidate the immune mechanisms involving several cell types and soluble factors released to injured tissue.

## Abbreviations

GCSF: granulocyte colony-stimulating factor
MYPS: myeloid phagocyte system
SDF-1: stromal derived factor-1
CXCR: C-X-C motif chemokine receptor
LXR: liver X receptor
IL: interleukin
MPS: mononuclear phagocyte system
M-CSF: macrophage-colony stimulating factor
Csf1: hematopoietic growth factor receptor
DC: dendritic cells
HSCs: hematopoietic stem cells
LPS: lipopolysaccharides
IFN: interferon
NK: natural killer
SOCS3: suppressor of cytokine signalling 3
TLR: toll-like receptor
TGF: transforming growth factor
ROS: reactive oxygen species
NETs: neutrophil extracellular traps
NE: neutrophil elastase
MPO: myeloperoxidase
BPI: bactericidal permeability increasing protein
MMP-9: matrix metalloproteinase-9
PAD4: peptidyl arginine deiminase 4
APC: antigen-presenting cells
T1DM: autoimmune type 1 diabetes mellitus
HLA: human leukocyte antigen
IL2R2: interleukin 2 receptor alpha
VNTR: insulin-variable number tandem repeat
VDR: vitamin D receptor
CTLA: cytotoxic T-lymphocyte-associated protein
CRP: C-reactive protein
PR3: proteinase 3
PMA: phorbol myristate acetate
CRAMP: cathelicidin-related antimicrobial peptide
RA: rheumatoid arthritis
PADI4: protein-arginine deiminase type 4
PAD: peptidyl arginine deiminase
ACPAs: autoantibodies to citrullinated protein antigens
CIA: collagen-induced arthritis
SF: synovial fluid
